# ENDOGENOUS EXCESS CORTISOL PRODUCTION AND DIABETES MELLITUS AS PREDISPOSING FACTORS FOR PULMONARY CRYPTOCOCCOSIS: A CASE REPORT AND LITERATURE REVIEW

**DOI:** 10.4103/0970-2113.45281

**Published:** 2008

**Authors:** B Thangakunam, D.J. Christopher, S Kurian, R Thomas, P James

**Affiliations:** Department of Pulmonary Medicine and Pathology *, Christian Medical College, Vellore, Tamil Nadu, India

**Keywords:** Pituitary, Cortisol, Cryptococcosis, Cushing's syndrome, Diabetes mellitus

## Abstract

Pulmonary cryptococcosis usually occurs as an opportunistic infection in immunocompromised patients. Endogenous Cushing's syndrome is associated with cortisol excess and can predispose to development of cryptococcal infections. We report a case of diabetic patient with ACTH secreting pituitary tumour who developed a cavitating lung mass. Computed tomography-guided biopsy of the lesion revealed mucicarminophilic budding forms of cryptococcus. Broncheoalveolar lavage culture grew Cryptococcus neoformans. There was radiological response to treatment with liposomal Amphotericin, but patient ultimately succumbed to septicemia and multiorgan failure. Opportunistic infections with organisms like Cryptococcus neoformans, should be considered in patients with endogenous Cushing's syndrome and a pulmonary infiltrate.

## INTRODUCTION

Pulmonary cryptococcosis occurs usually in the immunocompromised patients. Excess cortisol production can theoretically produce an immunodeficiency state and predispose to cryptococcosis. We describe a diabetic patient with ACTH-producing pituitary tumour who developed pulmonary cryptococcosis.

## CASE REPORT

A fifty-two year old bank manager, with history of systemic hypertension, Type II diabetes mellitus with nephropathy of eight years duration and ankylosing spondylosis of thirty-years duration, presented with history of decrease in vision and field defects in both eyes of two months duration. He had history of weight gain, proximal myopathy and easy bruisability. He subsequently developed altered behaviour in the form of irritability and periods of unresponsiveness. He was evaluated with MRI of brain, which showed a sellar mass (2.2x2x2.4 cm) with suprasellar extension extending into the right parasellar region encasing the cavernous segment of right internal carotid artery and impinging on optic chiasma (Fig I). Serum ACTH level at 8 am was 188 pg/mL (Normal 9–52pg/mL). Serum cortisol levels at 8 am and 6 pm was elevated, both being 72 µg/dL. His HIV serology was negative. His bone marrow examination was non-contributory.

**Fig 1:**
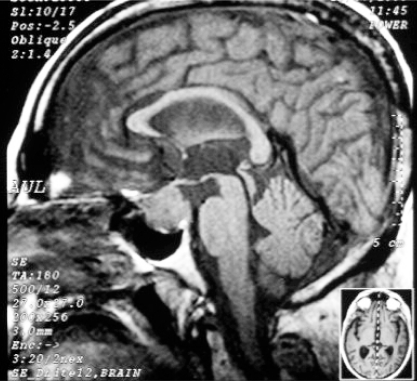
Sagittal section of MRI brain showing pituitary tumour

Subsequently, he developed breathing difficulty for which a chest radiograph was done which showed a cavitating mass in the posterior segment of left upper lobe (Fig II). Computed Tomography (CT) scan of chest confirmed the presence of this lesion and in addition revealed paratracheal and subcarinal lymphadenopathy. CT guided biopsy of the lung lesion was done, and it showed necrotic material infiltrated by few lymphocytes and mucicarminophilic budding forms of cryptococcus (Fig III). Fiberoptic bronchoscopy was normal and bronchoalveolar lavage culture grew Cryptococcus neoformans. In view of diabetic nephropathy, the patient was started on liposomal Amphotericin B (Ambisome) at a dose of 50mg on first day and then 150mg per day. He was unfit for surgical resection of the pituitary tumour. Ketoconazole at a dose of 400mg per day was started for control of hypercortisolism and continued for 22 days. Repeat chest radiograph after a cumulative dose of 950mg Ambisome showed decrease in size of the pulmonary lesion. However he developed Staphylococcus aureus bacteremia and multi-organ failure. He required endotracheal intubation and mechanical ventilation, on which he developed ventilator-associated pneumonia, refractory septic shock to which he succumbed.

**Fig 2:**
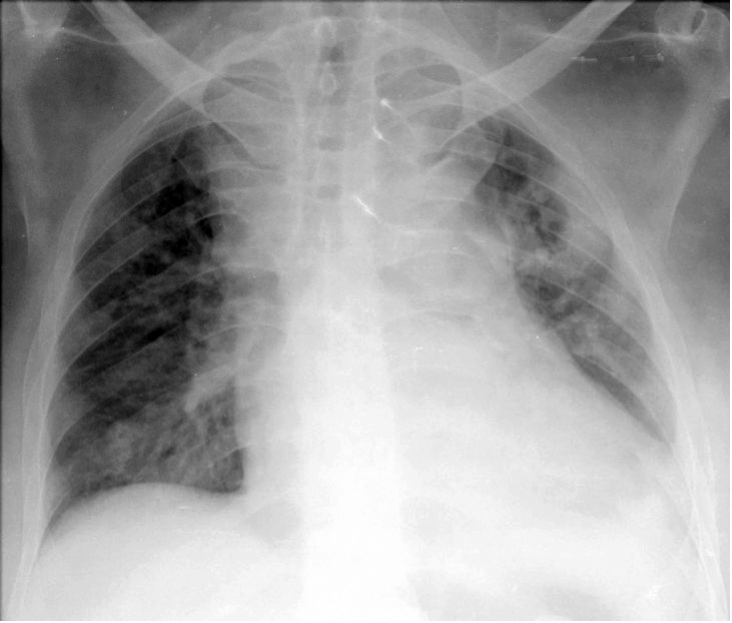
Chest radiograph showing thick walled cavity left upper lobe

**Fig 3:**
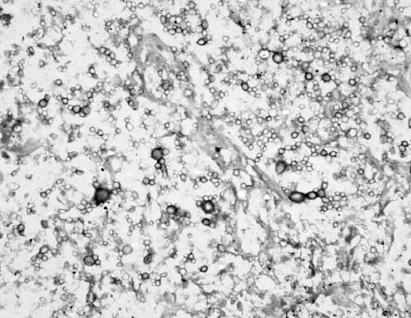
High power view of lung tissue with mucicarminophilic budding forms of cryptococcus

## DISCUSSION

Cryptococcus neoformans is a ubiquitous fungus, being commonly found in bird droppings, soil and decayed wood. It causes opportunistic infections, predominantly in patients with T-cell-mediated immune defects, like those with AIDS and transplant-related immunosuppression^1^,^2^,^3^. Corticosteroid therapy, cancer chemotherapy, diabetes mellitus and hematological malignancies are also predisposing factors for the development of cryptococcal infections. The spectrum of disease caused by this fungus ranges from asymptomatic pulmonary colonization to life-threatening meningitis and overwhelming cryptococcemia. Pulmonary cryptococcosis in non-immunocompromised patients is rare and most of such cases resolve spontaneously^3^.

In our patient the only risk factors for pulmonary cryptococcosis were endogenous Cushing's syndrome and diabetes mellitus. Endogenous Cushing's syndrome is associated with severe cortisol excess and can predispose to opportunistic infections. Pulmonary cryptococcosis in association with endogenous excess cortisol production is rare and only a few cases have been reported^4^–^7^ (Table No: I). In a case series of 143 patients with cryptococcosis who were HIV negative and not organ transplant recipients, only one patient had Cushing's disease as a risk factor^3^. Bakker et al described a patient with ACTH-dependent Cushing's syndrome whose clinical course was complicated by simultaneous infections with Pneumocystis carinii, Staphylococcus aureus, Candida albicans, Aspergillus fumigatus and Herpes simplex and which proved to be fatal^8^. Hypercortisolism associated with Cushing's syndrome appears to induce a transitory immune deficiency state and opens a window of opportunity for various infectious agents^9^.

**Table I:** Association of Pulmonary Cryptococcosis with Endogenous Cushing's syndrome reported in literature.

Author (year)	Pulmonary Cryptococcosis - Diagnostic modality	Type of Cushing's syndrome
Kramer M (1983)	-	ACTH dependent
Yamamoto N (1985)	-	ACTH dependent
Drew PA (1998)	FNAC	ACTH dependent
Takahashi S (2001)	BAL	Adrenal tumour
Lacativa PG (2004)	Necropsy	ACTH dependent

In non-AIDS patients cryptococcosis has also been associated with diabetes mellitus. In a series of 40 HIV-negative patients with cryptococcosis 14% had diabetes mellitus^10^. In another series of 22 patients with pulmonary cryptococcosis 7 had diabetes^11^.

The recommended treatment for non-meningeal cryptococcosis in HIV negative individuals is Amphotericin B in severe disease and Fluconazole in mild disease. In those with meningeal forms combination therapy with Amphotericin and Flucytosine is essential. In our patient cerebrospinal fluid examination to rule out meningeal involvement could not be done, due to raised intra cranial pressure.

In conclusion, patients with Cushing's syndrome or diabetes mellitus may be prone to infections including fungal infections such as with Cryptococcus neoformans. This should be kept in mind while evaluating such patients who develop pulmonary infections.
